# Analysis of the proteome of human airway epithelial secretions

**DOI:** 10.1186/1477-5956-9-4

**Published:** 2011-01-20

**Authors:** Mehboob Ali, Erik P Lillehoj, Yongsung Park, Yoshiyuki Kyo, K Chul Kim

**Affiliations:** 1 Department of Physiology and Lung Center, Temple University School of Medicine, Philadelphia, PA, USA; 2 Department of Pediatrics, University of Maryland School of Medicine, Baltimore, MD, USA; 3 Department of Pharmacology and Experimental Therapeutics, Thomas Jefferson University, 1020 Locust Street, JAH 364, Philadelphia, PA 19107, USA

## Abstract

**Background:**

Airway surface liquid, often referred to as mucus, is a thin layer of fluid covering the luminal surface that plays an important defensive role against foreign particles and chemicals entering the lungs. Airway mucus contains various macromolecules, the most abundant being mucin glycoproteins, which contribute to its defensive function. Airway epithelial cells cultured *in vitro *secrete mucins and nonmucin proteins from their apical surface that mimics mucus production *in vivo*. The current study was undertaken to identify the polypeptide constituents of human airway epithelial cell secretions to gain a better understanding of the protein composition of respiratory mucus.

**Results:**

Fifty-five proteins were identified in the high molecular weight fraction of apical secretions collected from *in vitro *cultures of well-differentiated primary human airway epithelial cells and isolated under physiological conditions. Among these were MUC1, MUC4, MUC5B, and MUC16 mucins. By proteomic analysis, the nonmucin proteins could be classified as inflammatory, anti-inflammatory, anti-oxidative, and/or anti-microbial.

**Conclusions:**

Because the majority of the nonmucin proteins possess molecular weights less than that selected for analysis, it is theoretically possible that they may associate with the high molecular weight and negatively charged mucins to form a highly ordered structural organization that is likely to be important for maintaining the proper defensive function of airway mucus.

## Background

Mucins are a heterogeneous group of polydisperse and high molecular weight O-glycosylated proteins, ranging from 300,000 to >1 × 10^7 ^Da [1]. Mucin genes encode polypeptide monomers that are synthesized as rod-shape apomucin cores and are post-translationally modified by exceptionally abundant and terminally sialylated glycan moieties. Mucins are secreted or inserted to the apical surface membrane of epithelial cells via a membrane-spanning hydrophobic domain [2,3]. Secreted mucins constitute the most abundant glycoprotein component of mucus, a gel-like substance that covers all wet-surfaced epithelia, including those of the respiratory, gastrointestinal, and genitourinary tracts [4-7]. Eighteen mucin (MUC) genes have been cloned, 12 of which are expressed in the respiratory tract [3]. Airway secreted mucins (MUC2, 5AC, 5B, 7, 8, 11, 13, 19, and 20) are produced by goblet cells on the surface epithelium and mucous cells from sub-mucosal glands [4,5,7]. Membrane-associated mucins in the airways include MUC1, MUC4, and MUC16. Mucin gene and protein structures, goblet cell mucin secretion and hypersecretion in airway diseases as well as the regulation of goblet cell hyperplasia have been well-documented in the literature [7-9]. However, the complex interactions between mucins and other protein components of airway mucus remain to be elucidated.

*In vitro *cultures of human and animal airway epithelial cells have been used to characterize mucin production under various experimental conditions. Human cell cultures from tracheal glands were reported to express a mixed serous and mucous phenotype [10,11]. Normal and cystic fibrosis cells from human tracheal glands isolated by the explant/outgrowth procedure secreted high molecular weight mucin-like glycoproteins and expressed the MUC2 gene [12]. The development and characterization of primary cell cultures from tracheal surface epithelium [13] that were highly enriched for secretory cells allowed the first identification of authentic airway mucins that were produced *in vitro *[14]. Confluent cultures of hamster tracheal epithelial cells cultured on a type I collagen gel synthesized and secreted high molecular weight glycoconjugates which eluted in the void volume upon Sepharose CL-4B column chromatography [14,15]. Biochemical analysis of these mucous glycoproteins showed both size and charge microheterogeneities that were remarkably similar to those of mucins produced *in vivo*.

The *in vitro *model of mucin production by airway epithelial cell cultures first established in hamsters was subsequently extended to guinea pig and human airway epithelium [16,17]. Proteomic studies have revealed that the protein composition of in vitro human airway epithelial cell secretions is particularly complex. Candiano *et al*. [18] used a two-dimensional SDS-PAGE/proteomics approach to identify the proteins secreted by *in vitro *cultures of polarized monolayers of human airway epithelial cells at resting and following stimulation with IL-4, IL-1β, TNF-α, or IFN-γ. Approximately 175 polypeptides were identified, among which were immune-related proteins, structural proteins, proteases, and protease inhibitors. Comparisons between treated and untreated conditions showed that the expression of several proteins was significantly modified by the different cytokines. Kesimer *et al*. [19] identified 134 proteins from apical secretions of primary airway epithelial cells, with 84 proteins (62.7%) being common with the proteins identified in *in vivo *human tracheobronchial sputum. Separation of the proteins by density gradient ultracentrifugation in the presence of 4 M guanidine hydrochloride identified 29 proteins that were present in the high-density, mucin-rich fraction. The nonmucin proteins in this fraction were also present in the lower-density pool, suggesting to the authors that they associated and copurified with the mucins. However, given that the proteins were isolated under denaturing conditions, the current study was conducted to identify mucin-associated proteins in *in vitro *human airway epithelial cell secretions isolated under more physiological conditions.

## Methods

### Preparation of human airway epithelial cell secretions (hAECS)

Primary normal human bronchial epithelial (NHBE) cells and culture media were purchased from MatTek Corp. (Ashland, MA). Air-liquid interface (ALI) cell cultures were maintained according to the manufacturer's protocol. The cells were seeded and incubated for 2 days at 37°C as submerged cultures before ALI culture was initiated. At confluence, the apical surface of the culture was washed with culture medium and the collected medium was referred to as hAECS. hAECS were collected daily for 5 days from multiple independent cultures of NHBE cells reflecting different cell donors and were pooled so that the data could not be attributed to artifacts of a single donor's genotype. Pooled samples were dialyzed against 100-fold volume of distilled water at 4°C for 2 days to approximately 5 mOsm/L, lyophilized, and stored at -80°C.

### Gel filtration chromatography

Approximately 1.80 mg of hAECS protein in 2.5 ml was separated on a Sepharose CL-4B gel filtration column (3.0 × 50 cm), pre-calculated for the void volume (V_0_) and total volume (V_t_) fractions with dextran blue and phenol red respectively. Proteins were applied and eluted in PBS, pH 7.2 with elution monitored by absorbance at 280 nm (A_280_). The V_0 _fractions containing high molecular weight mucins and associated proteins were collected, assayed for carbohydrate content by reaction with Periodic acid-Schiff reagents, and stored at -20°C until use.

### Periodic acid-Schiff carbohydrate assay

The Periodic acid-Schiff staining kit from Sigma (St. Louis, MO) was used to determine carbohydrate content according to the manufacturer's instructions. Briefly, 180 μl of sample was added to wells of a 96-well plate, followed by the addition of 20 μl of periodic acid solution, incubation at 37°C for 1 hr, addition of 20 μl of Schiff's reagent, and incubation at room temperature for 30 min. A_555 _was measured using a spectrophotometer.

### MUC5AC Westen blot assay

Samples were diluted in 1:4 in 40 mM Tris-acetate, pH 8.0 containing 40% glycerol and 0.8% bromophenol blue and heated at 100°C for 5 min. Samples were electrophoresed in 1.0% agarose gels (12 × 12 cm) using 40 mM Tris-acetate, pH 8.0 containing 1.0 mM EDTA and 0.1% SDS at 30 V for 20 hr at room temperature. After electrophoresis, the agarose gels were incubated with transfer buffer (0.06 M sodium citrate, pH 7.0, 0.6 M NaCl) for 30 min and proteins were transferred to PVDF membrane overnight by the capillary blotting. The membrane was blocked with PBS containing 0.5% Tween 20 for 1 hr, sequentially incubated overnight with primary anti-MUC5AC monoclonal antibody (1:2,000; 45 M1, GenWay Biotech, San Diego, CA), 1 hr with goat anti-mouse IgG secondary antibody (1:10,000; KPL, Gaithersburg, MD), and enhanced chemiluminescence substrate (Amersham GE Healthcare, Piscataway, NJ).

### Proteomics analysis

#### TCA precipitation of V_0 _fractions

V_0 _fractions were precipitated by addition of a 4-fold volume of TCA overnight at 4°C. Samples were centrifuged at 15,000 × g for 15 min at 4°C, the pellets were resuspended in 200 μl of acetone at -20°C, centrifuged, and air-dried.

#### One-dimensional SDS-polyacrylamide gel electrophoresis

TCA precipitated and acetone washed V_0 _fractions of hAECS were resuspended in a minimum volume of 30 mM Tris-HCl, pH 8.5-9.0 containing 2 M thiourea, 7 M urea, and 4% CHAPS. Thirty μg of protein was diluted in an equal volume of SDS-PAGE sample loading buffer (120 mM Tris-HCl, pH 6.8 containing 20% glycerol, 4% SDS, 200 mM DTT, and 0.01% bromophenol blue). Samples were resolved on one-dimensional 12% SDS-PAGE gels at 20 mA for 30 min and 50 mA until the bromophenol dye front reached the bottom of gel. Gels were silver-stained and photographed.

#### Nano-LC-IT MS

Gel slices obtained along the entire length of the SDS-PAGE gel were reduced with 10 mM DTT in 50 mM NH_4_HCO_3 _for 30 min at 37°C and alkylated with 50 mM iodoacetamide in 50 mM NH_4_HCO_3 _for 30 min at room temperature in the dark. Gel slices were destained with 50% (v/v) acetonitrile in 50 mM NH_4_HCO_3_, dehydrated with acetonitrile, and 40 ml of 12.5 mg/ml trypsin in 50 mM NH_4_HCO_3 _was added to cover the gel pieces. Following overnight trypsin digestion, peptides released into solution were desalted, dried by vacuum centrifugation, and resolubilized in 30 ml of 0.1% (vol/vol) trifluoroacetic acid. The sample was loaded onto a 2 mg capacity peptide trap (CapTrap; Michrom Bioresources, Auburn, CA) prior to separation on a C-18 reversed-phase HPLC capillary column (15 cm, 75 mm, Agilent, Santa Clara, CA) at 300 nl/min delivered by an Agilent 1100 LC pump. A mobile-phase gradient was run using mobile phases A (1% acetonitrile/0.1% formic acid) and B (80% acetonitrile/0.1% formic acid) from 0 to 10 min with 0-15% B, 10-60 min with 15-60% B, and 60-65 min with 60-100% B. Eluted peptides were analyzed by nanoelectrospray ionization (ESI) tandem mass spectrometry (MS) using a HCT Ultra ion trap mass spectrometer (Bruker Daltonics Inc., Billerica, MA). ESI was delivered using distal-coating spray Silica tip (ID 20 mM, tip inner ID 10 mM, New Objective, Woburn, MA) at a spray voltage of -1300 V. Using automatic switching between MS and MS/MS modes, MS/MS fragmentation was performed on the two most abundant ions on each spectrum using collision-induced dissociation with active exclusion (excluded after two spectra and released after 2 min). The entire system was controlled by HyStar 3.1 software.

#### Data analysis

Mass spectra processing was performed with Data Analysis 3.3 software and the generated peak list was submitted to an in-house Mascot (Matrix Science, Boston, MA) search engine (version 2.2) for searching against the Swiss-Prot protein database (version 56.6 containing 410,518 sequences). Mascot search parameters were set as follows: taxonomy human, trypsin enzyme with maximal 1 missed cleavage, cysteine carboxymethylation as fixed modification, methionine oxidation as variable modifications, and 0.30 Da mass tolerance for precursor peptide ions and 0.4 Da for MS/MS fragment ions. All peptides matches were filtered with an ion score cutoff of 10. The following criteria were used for protein identification: one peptide with ion score 50 or higher, two or more peptides with one ion score higher than 31 (p < 0.05) and one ion score 20 or higher, and molecular weight match between theoretical and experimental values applied for proteins with Mascot scores less than 70. Protein identifications were only accepted and reported as distinct hits if they were based on at least one unique peptide that passed probability validation. Under these criteria, no false positive matches were found when searched against a reversed-decoy Swiss-Prot database. Identified proteins were analyzed for molecular and biological function using the Panther protein database [20] and literature search using PubMed.

## Results

### Gel filtration chromatography of hAECS

**Figure 1:**
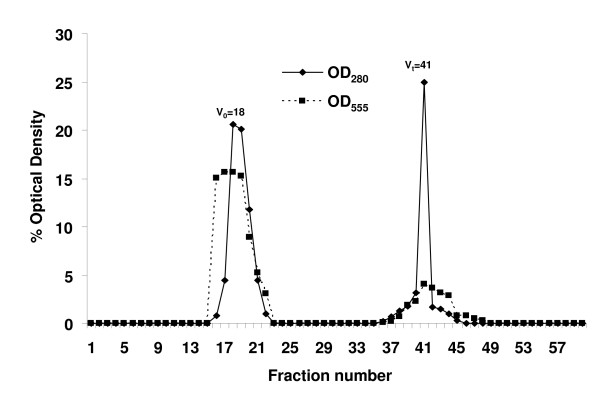
**Sepharose CL-4B gel filtration chromatography of hAECS**. The percent optical densities at 280 nm (solid line) and 555 nm (dashed line) are indicated on the x-axis while individual column fractions are indicated on the y-axis. The void volume (V_0_) and total volume (V_t_) are indicated.

Figure 1 shows the Sepharose CL-4B gel filtration profile of hAECS collected from the apical surface of *in vitro *cultures of primary NHBE cells cultured at an ALI. Two peaks of protein elution were detected by A_280_, the V_0 _(fraction 18) and the V_t _(fraction 41) (denoted by solid line). The periodic acid-Schiff assay confirmed the presence of glycoproteins in the V_0 _fractions (denoted by dotted line).

### Gel electrophoretic analysis of hAECS proteins

**Figure 2:**
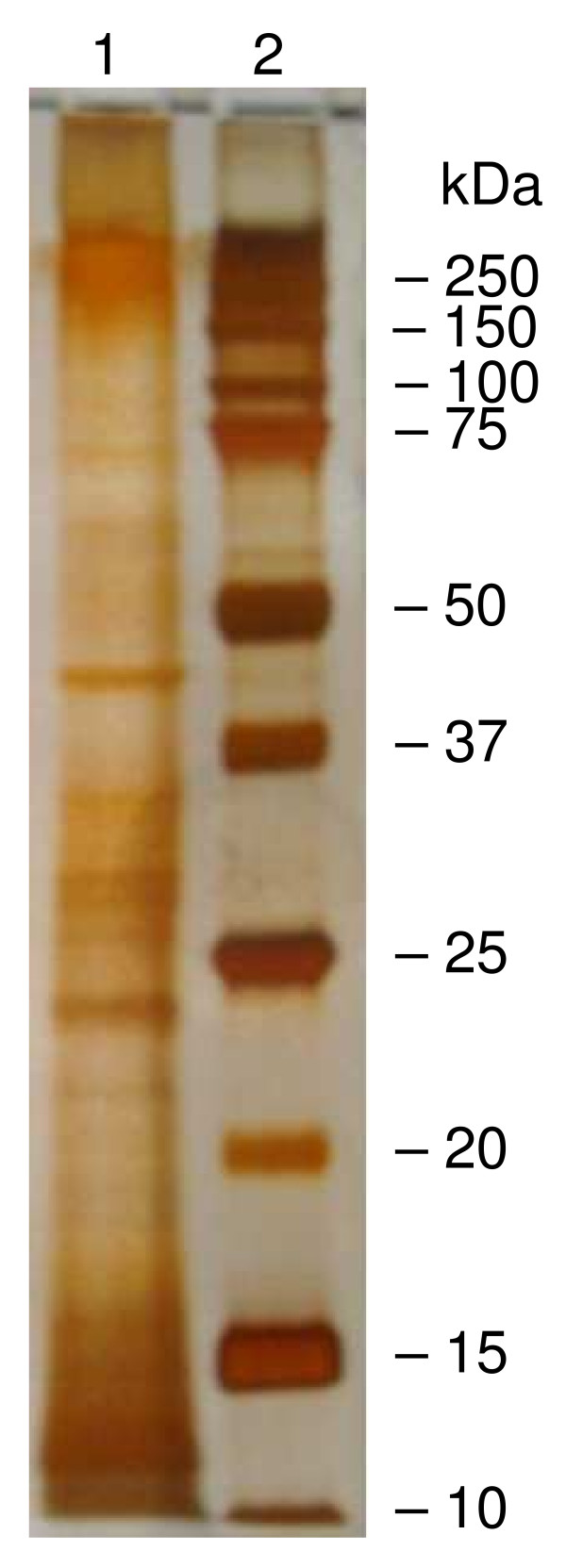
**One-dimensional SDS-polyacrylamide gel electrophoresis of V**_**0 **_**fraction from gel filtration chromatography**. Proteins were separated on a 12% gel under reducing conditions and visualized by silver staining. Lane 1, hAECS sample. Lane 2, prestained molecular weight marker proteins. Molecular weights in kDa are indicated on the right.

Silver staining of hAECS proteins in the Sepharose CL-4B V_0 _fraction that were resolved by one-dimensional SDS-PAGE showed the presence of multiple protein bands (Figure 2). Most of the proteins bands were <75 kDa with a diffuse area of staining at >250 kDa.

### Protein identification by nano-LC-IT MS

**Figure 3:**
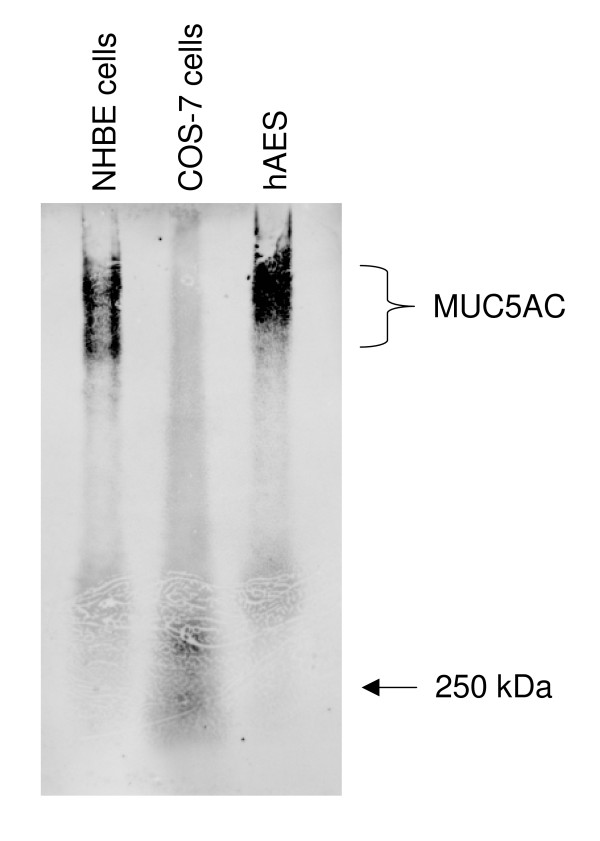
**MUC5AC Western blot analysis**. Equal protein aliquots (100 μg) of lysates of NHBE cells, COS-7 cells (negative control), or hAECS from NHBE cells were resolved by agarose gel electrophoresis, transferred to PVDF membrane, and reacted with anti-MUC5AC antibody (45M1). The positions of MUC5AC and the 250 kDa marker protein are shown on the right.

**Figure 4:**
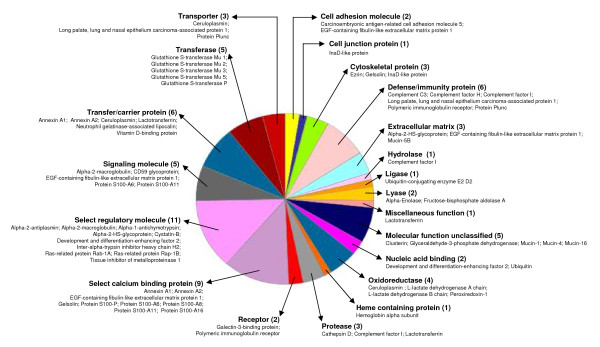
**Pie chart of molecular functions of proteins in high molecular weight hAECS identified by nano-LC-IT MS**. Proteins are listed by category, number of proteins in the category and specific proteins identified.

**Figure 5:**
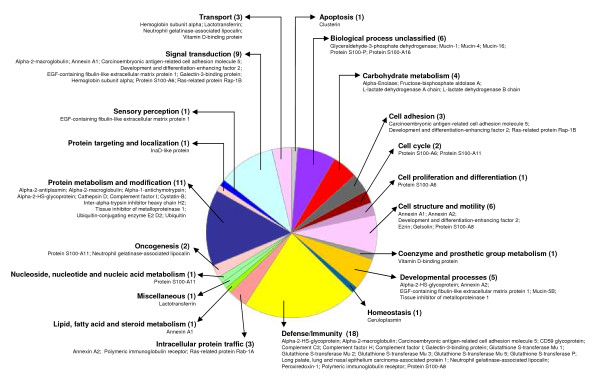
**Pie chart of biological functions of proteins in high molecular weight hAECS identified by nano-LC-IT MS**. Proteins are listed by category, number of proteins in the category and specific proteins identified.

**Figure 6:**
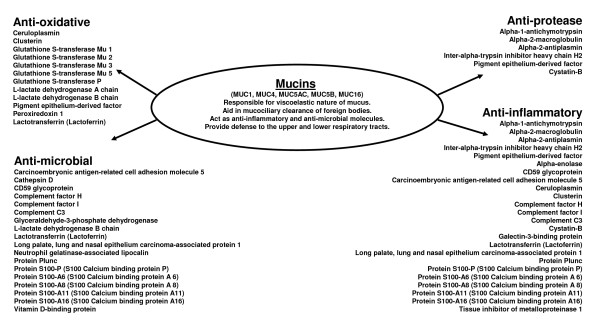
**Categorization of proteins in high molecular weight hAECS identified by nano-LC-IT MS**.

The hAECS sample resolved by gel filtration chromatography and SDS-PAGE was divided into ten 0.5 cm gel slices, each of which was processed as described in the Methods. Fifty-five proteins were identified (Table 1). Among these were MUC1, MUC4, MUC5B, and MUC16 in the high molecular weight region (>250 kDa) of the acrylamide gel. While MUC5AC was not identified by proteome analysis, this mucin was readily detected by Western blotting of hAECS (Figure 3). The molecular (Figure 4) and biological (Figure 5) functions of the identified proteins were made using the Panther protein database search engine. According to molecular function analysis, most of the identified proteins were classified as regulatory molecules (20.0%), calcium binding proteins (16.4%), transfer/carrier proteins (10.9%), and defense/immunity-related proteins (10.9%). According to biological function analysis, the majority of the identified proteins belonged to the defense/immunity (32.7%), protein metabolism and modification (23.6%), and signal transduction (16.4%) categories. Based on a combined search of the published literature and the Panther protein database analysis, the identified proteins were grouped into 4 major functional subgroups, namely inflammatory, anti-inflammatory, anti-oxidant, and anti-microbial (Figure 6).

**Table 1:** Proteins identified in the V_0 _fraction of hAECS following SDS-PAGE.

Gene Name	Protein Name	**Gel Slice**^**a**^	**Ion Score**^**b**^	**Mol Wt (Da)**^**c**^	**Matches**^**d**^
A2AP_HUMAN	Alpha-2-antiplasmin	3-4	57	54,531	1
A2MG_HUMAN	Alpha-2-macroglobulin	2	134	163,175	3
AACT_HUMAN	Alpha-1-antichymotrypsin	4	240	47,621	6
ANXA1_HUMAN	Annexin A1	5	245	38,690	7
ANXA2_HUMAN	Annexin A2	5	539	38,580	14
CEAM5_HUMAN	Carcinoembryonic antigen-related cell adhesion molecule 5	2-3	75	76,748	1
CATD_HUMAN	Cathepsin D	4	102	44,524	6
CD59_HUMAN	CD59 glycoprotein	9-10	121	14,168	5
CERU_HUMAN	Ceruloplasmin	2	85	122,128	3
CLUS_HUMAN	Clusterin	3-4	167	52,461	3
CFAH_HUMAN	Complement factor H	2	58	139,005	1
CFAI_HUMAN	Complement factor I	3	64	65,677	1
CO3_HUMAN	Complement C3	1	1,453	187,030	38
CYTB_HUMAN	Cystatin-B	10	91	11,133	2
DDEF2_HUMAN	Development and differentiation-enhancing factor 2	2	45	111,581	2
ENOA_HUMAN	Alpha-enolase (Enolase 1)	4	110	47,139	2
EZRI_HUMAN	Ezrin	3	105	69,370	2
FBLN3_HUMAN	EGF-containing fibulin-like extracellular matrix protein 1	3-4	39	54,604	1
FETUA_HUMAN	Alpha-2-HS-glycoprotein (Fetuin A)	5	142	39,300	3
ALDOA_HUMAN	Fructose-bisphosphate aldolase A	5	76	39,395	1
LG3BP_HUMAN	Galectin-3-binding protein	3	120	65,289	5
GELS_HUMAN	Gelsolin	2	293	85,644	10
G3P_HUMAN	Glyceraldehyde-3-phosphate dehydrogenase	5	187	36,030	4
GSTM1_HUMAN	Glutathione S-transferase Mu 1	6	128	25,695	6
GSTM2_HUMAN	Glutathione S-transferase Mu 2	6	168	25,728	6
GSTM3_HUMAN	Glutathione S-transferase Mu 3	6	180	26,542	9
GSTM5_HUMAN	Glutathione S-transferase Mu 5	6	172	25,658	7
GSTMP1_HUMAN	Glutathione S-transferase P	6-7	53	23,341	1
HBA_HUMAN	Hemoglobin subunit alpha	9	56	15,248	1
INADL_HUMAN	InaD-like protein	1	53	196,247	1
ITIH2_HUMAN	Inter-alpha-trypsin inhibitor heavy chain H2	2	112	106,370	2
LDHA_HUMAN	L-lactate dehydrogenase A chain	5	131	36,665	3
LDHB_HUMAN	L-lactate dehydrogenase B chain	5	114	36,615	2
TRFL_HUMAN	Lactotransferrin (Lactoferrin)	2	100	78,132	7
LPLC1_HUMAN	Long palate, lung and nasal epithelium clone protein 1	3-4	1,188	52,408	36
MUC1_HUMAN	Mucin-1	1	131	121,999	3
MUC4_HUMAN	Mucin-4	1	131	231,440	3
MUC5B_HUMAN	Mucin-5B	1	1,207	590,122	27
MUC16_HUMAN	Mucin-16	1	56	2,351,813	2
NGAL_HUMAN	Neutrophil gelatinase-associated lipocalin	7	344	22,574	12
PRDX1_HUMAN	Peroxiredoxin-1	7	125	22,096	4
PEDF_HUMAN	Pigment epithelium-derived factor	4	187	46,313	5
PIGR_HUMAN	Polymeric immunoglobulin receptor	2	999	83,232	24
PLUNC_HUMAN	Protein PLUNC	6	438	26,696	13
S100P_HUMAN	Protein S100-P (S100 calcium binding protein P)	10	118	10,393	3
S10A6_HUMAN	Protein S100-A6 (S100 calcium binding protein A6)	10	48	10,173	2
S10A8_HUMAN	Protein S100-A8 (S100 calcium binding protein A8)	10	61	10,828	2
S10AB_HUMAN	Protein S100-A11 (S100 calcium binding protein A11)	10	41	11,733	1
S10AG_HUMAN	Protein S100-A16 (S100 calcium binding protein A16)	10	42	11,794	1
RAB1A_HUMAN	Ras-related protein Rab-1A	7-8	100	22,663	2
RAB1B_HUMAN	Ras-related protein Rap-1B	7-8	118	20,812	3
TIMP1_HUMAN	Tissue inhibitor of metalloproteinase 1	7	74	23,156	1
UB2D2_HUMAN	Ubiquitin-conjugating enzyme E2 D2	9	40	16,724	2
UB1Q_HUMAN	Ubiquitin (ribosomal protein S27a)	10	154	8,560	3
VTDB_HUMAN	Vitamin D-binding protein	3-4	142	52,929	2

^a^SDS-PAGE gel slice number (1 [top] - 10 [bottom]) from which the protein was isolated.

^b^Ion Score, probability value that an observed peptide match is a true match; cuttoff value = 10.

^c^Theoretical molecular weight from Swiss-Prot database.

^d^Number of tryptic peptides generated that match the mass of theoretical trypsin peptides.

## Discussion

Among the 55 proteins that were identified in the high molecular weight fraction of hAECS from *in vitro *cultures of primary NHBE cells collected under physiological conditions, 43 (78.2%) were unique to this study and 12 (21.8%) were common to proteins identified in the mucin-rich apical secretions isolated under denaturing conditions by Kesimer *et al*. [19]. The shared proteins of the two studies are: MUC1, MUC4, MUC5B, MUC16, CD59, complement C3, clusterin, glutathione S-transferase, lactotransferrin, long palate, PLUNC (palate, lung and nasal epithelial clone) protein, LPLUNC1 (long PLUNC), and the polymeric immunoglobulin receptor. All of the proteins unique to our study comprise important inflammatory, anti-inflammatory, anti-microbial and/or anti-oxidant components that are elicited by the respiratory tract in response to exposure to infectious or injurious insults [21]. On the other hand, among the proteins that were identified by Kesimer *et al*. [19] but not in this study are alpha-defensin 1 and secretory leukocyte peptidase inhibitor (SLPI), both of which are thought to have broad-spectrum antibiotic activities [22].

While it is tempting to speculate that the proteins we identified may form a physiologically relevant association with mucins, it is important to emphasize that they may or may not be actively secreted by the cells, or may simply constitute membrane-bound proteins that passively adhere to the mucins, although other membrane components, such as lipids, were not identified. Further, given that the apical washings were dialyzed against distilled water, we cannot exclude the possibility that some of the identified polypeptides are intracellular proteins from sloughed cells and thus are not necessarily components of airway mucus. We also cannot rule out that our results may not reflect the proteome of *in vivo *airway lining fluid given the probable contributions of other cell types and blood-borne proteins that were not present in the NHBE cell cultures. However, the contribution of the cell culture media appears negligible since serum albumin, the most abundant protein in culture media, was not identified in the current analysis.

The presence of MUC1, MUC4, MUC5B, and MUC16 in *in vitro *hAECS confirms prior studies of mucin glycoprotein secretion by human and animal airway epithelial cell cultures [13-19]. Mucins constitute the main protein components of mucus and provide a multifaceted function to the mucosal surface from which they are produced, ranging from lubrication to cell signaling to forming protective physical barriers against chemical or biological damage [4-6]. But whether or not these functions are provided by mucins alone or in association with other proteins remains an unanswered question, which we and others [18,19] have attempted to address. Specifically, several reports have promoted the concept that the association between mucins and other proteins plays a vital role in defense of the respiratory and gastrointestinal mucosa against microbial infections [23-25], including an early model put forth for mucin-protein interactions in lung mucus by Rose *et al*. [26].

The inability to identify MUC5AC mucin by proteomic analysis may have been due to insensitivity of the detection instrumentation, incomplete trypsin digestion, a relatively low abundance of this protein, and/or failure of TCA to precipitate MUC5AC. Other proteomic studies of hAECS collected from *in vitro *cell cultures have demonstrated that MUC5B is the predominant mucin with much lesser amounts of MUC5AC [19]. By microarray hybridization analysis, expression of MUC1 and MUC5B mucins, but not MUC5AC, were up-regulated during ALI culture of human bronchial epithelial cells [27]. Therefore, these or other technical artifacts are the most likely explanation for the current result given that MUC5AC was clearly present in the hAECS by Western blot analysis and the fact that our prior study identified human MUC5AC in the spent culture medium of A549 lung epithelial cells and rat Muc5ac in apical washings of primary tracheal epithelial cell cultures at an ALI [28].

The family of proteins identified in this study were categorized according to molecular and biological functions by Pather analysis. According to the original definitions of Gene Ontology by Ashburner *et al*. [29], molecular function is defined as the elemental activity of a gene product at the molecular level, such as binding or catalysis, while a biological function refers to the collected operations or sets of molecular events with a defined beginning and end, and pertinent to the functioning of integrated living units (cells, tissues, organs, and organisms). A biological function is accomplished via one or more ordered assemblies of molecular functions. In this study, the molecular functions associated with the greatest number of proteins that were identified in the high molecular weight fraction of the hAECS samples were regulatory (11 proteins), calcium binding (9 proteins), transfer/carrier (6 proteins), and defense/immunity (6 proteins). The biological functions associated with the greatest number of proteins were defense/immunity (18 proteins), protein metabolism and modification (11 proteins), and signal transduction (9 proteins). As noted above, some of these functions have been previously ascribed to airway mucins.

In addition, this collection of proteins could be further classified into inflammatory, anti-inflammatory, anti-oxidative, and anti-microbial categories based upon search of the published literature and molecular/biological function analyses. Some of the proteins fall into more than one category because they perform more than one function. For example, protease inhibitors may act both as inflammatory and anti-inflammatory components of mucus, depending upon the context of their expression [21]. Protease inhibitors that were identified in the present study were alpha-1-antichymotrypsin, alpha-2-macroglobulin, alpha-2-antiplasmin, inter-alpha-trypsin inhibitor heavy chain H2, pigment epithelium-derived factor, cystatin-B, and tissue inhibitor of metalloproteinase 1. Many of these proteins have been shown to regulate protease activities in various diseases that afflict airway epithelia [30-35].

The anti-oxidative proteins identified herein were ceruloplasmin, clusterin, glutathione S-transferase (GST), pigment epithelium-derived factor, peroxiredoxin 1 (Prdx1), lactate dehydrogenase, lactotransferrin, ubiquitin, and ubiquitin-conjugating enzyme E2 D2. Amongst these, GST and Prdx1 are probably the best characterized in the respiratory tract. GST provides redox balance in response to production of reactive oxygen species and has been reported to be involved in host defense in the lung [36]. The Prdx family of anti-oxidant enzymes reduces peroxides, lipid hydroperoxides and peroxynitrites, and the role of Prdx1 in airway inflammation has been reviewed [37]. Within the group of anti-microbial proteins, CD59 is involved in inflammatory signal transduction in T cells in response to pathogenic insults [38], while components of the complement system act as antibiotic components during host defense against bacteria that commonly infect the respiratory tract, including *Pseudomonas aeruginosa*, *Klebsiella pneumoniae*, and *Streptococcus pneumoniae *[39-41]. The palate, lung and nasal epithelial clone (PLUNC) family can be subdivided into short (SPLUNC) and long (LPLUNC) proteins [42]. In this classification, the original protein called PLUNC is now SPLUNC1, which is expressed in sub-mucosal glands of normal individuals and expression is increased in cystic fibrosis lungs, especially in the surface epithelia of the conducting airways. PLUNC was reported to protect the airway epithelial sodium channel (ENaC) from proteolytic cleavage, to prevent respiratory infections, and to inhibit lung allergic responses [43,44]. Finally, the S100 series of Ca^2+ ^binding proteins are highly expressed in neutrophils and monocytes [45,46] and have been reported as anti-microbial agents against *P. aeruginosa *[47] as well as novel TLR ligands important in modulating inflammation [45,48].

## Conclusion

We identified 55 proteins that were present in the high molecular weight fraction of *in vitro *human airway epithelial cell apical secretions isolated under physiological conditions. Among the mucin gene products that were identified were MUC1, MUC4, MUC5B, and MUC16. By proteomic analysis, these proteins could be classified as inflammatory, anti-inflammatory, anti-oxidative, and/or anti-microbial. Given that the majority of these proteins in their monomeric form possess molecular weights far less than that selected for analysis, it is theoretically possible that they may associate with the highly sialylated and negatively charged mucins through ionic and hydrophobic interactions to form a highly ordered structural organization that may be important for maintaining the proper function of airway mucus.

## List of abbreviations used

ALI: air-liquid interface; ESI: electrospray ionization; hAECS: human airway epithelial cell secretion; kDa: kiloDalton; MS: mass spectrometry; Mol Wt: molecular weight; MUC: mucin; NHBE: normal human bronchial epithelial; SDS-PAGE: sodium dodecyl sulfate-polyacrylamide gel electrophoresis

## Competing interests

The authors declare that they have no competing interests.

## Authors' contributions

MA carried out the proteomic analysis and drafted the manuscript. EPL conducted preliminary experiments and wrote the final manuscript. YP and YH performed the cell culture and prepared the samples. KCK conceived of the study, obtained grant funding, and participated in experimental design and coordination. All authors read and approved the final manuscript.
